# Positive Imagery-Based Cognitive Bias Modification as a Web-Based Treatment Tool for Depressed Adults: A Randomized Controlled Trial

**DOI:** 10.1177/2167702614560746

**Published:** 2015-01

**Authors:** Simon E. Blackwell, Michael Browning, Andrew Mathews, Arnaud Pictet, James Welch, Jim Davies, Peter Watson, John R. Geddes, Emily A. Holmes

**Affiliations:** 1 Medical Research Council Cognition and Brain Sciences Unit; 2 Department of Psychiatry, University of Oxford; 3 Department of Psychology, University of California, Davis; 4 Institute of Psychiatry, King’s College London; 5 Department of Psychology, University of Geneva; 6 Department of Computer Science, University of Oxford

**Keywords:** depression, cognitive-bias modification, Internet health, mental imagery, cognitive therapy, anhedonia

## Abstract

Depression is a global health problem requiring treatment innovation. Targeting neglected cognitive aspects may provide a useful route. We tested a cognitive-training paradigm using positive mental imagery (imagery cognitive bias modification, imagery CBM), developed via experimental psychopathology studies, in a randomized controlled trial. Training was delivered via the Internet to 150 individuals with current major depression. Unexpectedly, there was no significant advantage for imagery CBM compared with a closely matched control for depression symptoms as a whole in the full sample. In exploratory analyses, compared with the control, imagery CBM significantly improved anhedonia over the intervention and improved depression symptoms as a whole for those participants with fewer than five episodes of depression and those who engaged to a threshold level of imagery. Results suggest avenues for improving imagery CBM to inform low-intensity treatment tools for depression. Anhedonia may be a useful treatment target for future work.

Many treatments have been developed for depression, but people are often unable to access them, and even if they can, the treatment may be ineffective: Even our best (psychological or pharmacological) are successful for only about 50% of depressed individuals (Hollon, Stewart, & Strunk, 2006). There is increasing recognition that to reduce the huge global burden of depression (World Health Organization, 2008), we need to develop interventions that are not only more accessible (e.g., Internet-delivered psychological therapies; Andersson, Riper, & Carlbring, 2014) but also more effective and resource efficient (Kazdin & Rabbitt, 2013). A key strategy is the identification and targeting of clinical mechanisms that current treatments fail to improve (e.g., Insel, 2012) and greater personalization of treatments (e.g., Fu, Steiner, & Costafreda, 2013; Johansson et al., 2012; Kazdin & Blase, 2011). Cognitive science offers one route to develop more effective, targeted treatment tools by identifying both the key cognitive mechanisms involved in the maintenance of a disorder and a potential means to modify them.

In depression, promising targets for intervention are offered by the cognitive biases that characterize the disorder—dysfunctional patterns in interpretation, attention, and memory (e.g., Gotlib & Joormann, 2010; Mathews & MacLeod, 2005). These have been the target of cognitive-training paradigms referred to as cognitive bias modification (CBM), which aim to retrain dysfunctional biases and thus improve symptoms (Koster, Fox, & MacLeod, 2009). Although in its early stages, interest in clinical applications of CBM paradigms has accelerated in recent years, with new domains of disorder and populations coming under investigation (Lau, 2013; Woud & Becker, 2014).

We have developed a specific CBM paradigm as a potential treatment tool or adjunct for use in depression that focuses on two cognitive targets: mental imagery and interpretation. This “imagery CBM” involves repeated practice in generating positive resolutions via mental imagery when confronted with ambiguous stimuli, with the aim of instilling a more adaptive bias to automatically imagine positive resolutions of novel ambiguous information in everyday life (e.g., Holmes, Mathews, Dalgleish, & Mackintosh, 2006; Holmes, Mathews, Mackintosh, & Dalgleish, 2008). The paradigm emerged from the experimental literature on modification of negative interpretive biases (Mathews & Mackintosh, 2000), and in its development for depression, there has been increasing emphasis on the mental imagery component (Holmes, Lang, & Deeprose, 2009). Depression has been associated with a deficit in positive future imagery (Holmes, Lang, Moulds, & Steele, 2008; Morina, Deeprose, Pusowski, Schmid, & Holmes, 2011). Thus, depressed individuals may particularly benefit from the repeated practice in generating positive mental images that is encouraged by imagery CBM. Furthermore, mental imagery has been relatively neglected in depression and, thus, may provide a new and promising avenue for treatment development (Weßlau & Steil, 2014).

A series of experimental studies with healthy participants (Holmes, Coughtrey, & Connor, 2008; Holmes et al., 2006; Holmes, Lang, & Shah, 2009; Holmes & Mathews, 2005; Nelis, Vanbrabant, Holmes, & Raes, 2012) and dysphoric individuals (Pictet, Coughtrey, Mathews, & Holmes, 2011) established the effects of imagery CBM in modifying interpretive bias and increasing positive affect in the laboratory. Furthermore, they demonstrated the importance of the instructions to use mental imagery (as opposed to verbal processing) for these effects. A single case series of imagery CBM completed daily for 1 week by seven depressed participants in their own homes provided initial evidence of clinical efficacy in reducing symptoms of depression (Blackwell & Holmes, 2010). Two subsequent preliminary randomized clinical studies (*n* = 13 per condition, again home-based) demonstrated a greater reduction in symptoms of depression when imagery CBM was compared with a control condition (Lang, Blackwell, Harmer, Davison, & Holmes, 2012; Torkan et al., 2014). Williams, Blackwell, Mackenzie, Holmes, and Andrews (2013) investigated a combination therapy of imagery CBM, delivered via the Internet, followed by Internet-based cognitive behavior therapy (iCBT). The combined intervention (*n* = 38) was compared with a wait list (*n* = 31) and showed a significant benefit on symptoms of depression after both the imagery CBM and the iCBT components.

Our aim in the current study was to build on these results and further develop imagery CBM “from the lab toward the clinic” as a potential low-intensity treatment tool for depression. We extended the cognitive-training schedule from 1 to 4 weeks, doubling the number of training sessions completed at home from 6 to 12, and developed an Internet-delivered version. We also extended the follow-up from 2 weeks to 6 months. We aimed to recruit a larger sample of participants with current major depression than used in previous studies, and to carry out the study within the more rigorous evaluative framework of a randomized controlled trial (RCT) to test the potential clinical efficacy of the imagery CBM in reducing symptoms of depression.

The trial also allowed us to examine mechanisms such as specific cognitive processes and symptoms influenced by imagery CBM. For example, the positive mental imagery aspect of the intervention suggests one potential clinical target that had previously not been explored: anhedonia. Anhedonia, the loss of interest in or enjoyment from activities, constitutes, alongside depressed mood itself, one of the two core diagnostic features of depression (American Psychiatric Association, 2013). However, anhedonia does not respond well to current first-line treatments (pharmacological or psychological) and is predictive of poorer treatment outcomes (Treadway & Zald, 2011; Uher et al., 2012). Imagery CBM involves repeatedly imagining oneself engaging in a broad range of activities that resolve with positive outcomes (e.g., going to work, meeting a friend, getting up in the morning). Many of the training scenarios specify one or more positive emotions to be imagined, for example, enjoyment, pleasure, interest, or excitement. The repeated practice in imagining, enjoying, and gaining pleasure from activities during the imagery CBM may therefore lead to an increased ability to anticipate positive emotional outcomes from activities in daily life, something that may be particularly problematic in depression (Dunn, 2012; Sherdell, Waugh, & Gotlib, 2012).

Early indicators of how to match patients to treatments, or patient stratification (Fu et al., 2013), are also important in development of experimental treatments. Potential treatment responders may be identified as falling into a particular clinical subgroup, or, alternatively, there may be more subtle indicators of patient engagement with the intervention that may be identifiable, such as early performance on a task (e.g., Clarke, Chen, & Guastella, 2012; Eberl et al., 2013). There may be large variation in treatment adherence and fidelity in Internet-delivered interventions (cf. Christensen, Griffiths, & Farrer, 2009), and studies have been criticized for the failure to investigate this (Kiluk et al., 2011). We therefore sought to assess such aspects in the current study.

This article first focuses on the main outcome of the trial, change in symptoms of depression, and then addresses other outcome and process variables relevant to understanding the transition from experimental psychopathology research to a clinical trials framework. Our primary hypothesis^1^ was that participants who completed the imagery CBM intervention (imagery condition) would demonstrate a greater decrease in symptoms of depression during the 4 weeks from baseline to posttreatment than would participants completing a closely matched (i.e., active) control version of the program (control condition). Secondary hypotheses were that there would be greater improvements in cognitive targets from baseline to posttreatment and that improvements would be maintained at 1-, 3-, and 6-month follow-up. Consistent with our interest in positive imagery and emotion, in further exploratory analyses we investigated the effect of imagery CBM on anhedonia. We also examined for whom (on the basis of baseline measures) the imagery CBM may be more effective and the role of active engagement in the training on outcomes.

## Method

### Study design and participants

We conducted an RCT with two parallel groups. Recruitment was via advertisement in local media (newspapers, radio), Web sites (e.g., Google, Facebook), and community, university, and health settings in the local area.

Eligible participants were those aged 18 to 65 who were fluent in written and spoken English and who met *Diagnostic and Statistical Manual of Mental Disorders* (4th ed., text rev.; American Psychiatric Association, 2000) criteria for a current major depressive episode assessed via a semi-structured clinical interview (the Structured Clinical Interview for *DSM–IV–TR* Axis I Disorders, SCID; First, Spitzer, Gibbon, & Williams, 2002). In addition, participants had to be able to give informed consent, to access the Internet-based intervention, and to attend the research center for assessment appointments. We excluded participants who met criteria for a current psychotic or substance-abuse disorder, had a history of mania or hypomania, had started or changed dose of antidepressant medication during the past month, were currently receiving psychological therapy, or were involved in other current treatment trials.

Entrance to the trial was via self-referral. On contacting the research team, potential participants completed a set of screening questionnaires, including demographics and the Beck Depression Inventory–II (BDI-II; Beck, Steer, & Brown, 1996), via the study Web site. Participants scoring 14 or above on the BDI-II were invited for the eligibility assessment. In July 2012, we amended the protocol to include a brief structured telephone screening interview for all participants who scored 14 or above on the BDI-II, which was designed to screen out those participants who obviously met exclusion criteria.

The eligibility assessment took place at the research center. Participants gave written informed consent, after which a researcher conducted the SCID interview and collected information about current and past treatments for mental health. Ineligible participants were debriefed and provided with information about accessing local mental-health services, if appropriate. Eligible participants were invited to return for a face-to-face baseline assessment at the research center (see the Trial Sites and Approvals section in the appendix).

Ethical approval was provided by the National Research Ethics Service (NRES) Committee South Central–Oxford C (11/SC/0278). The study was prospectively registered (clinicaltrials.gov identifier NCT01443234).

### Intervention

The imagery CBM intervention comprised 12 sessions completed at home via the study Web site during a 4-week period. Six sessions were in an auditory form in which participants listened to audio recordings of descriptions of everyday situations (approximately 10 s each) and were instructed to imagine themselves in the scenarios “as if actively involved, seeing them through your own eyes” (Holmes et al., 2006). As in previous studies, the descriptions were initially ambiguous as to their resolution but always ended positively. The other 6 sessions used stimuli in a picture-word form in which participants were presented with ambiguous photos of mostly everyday scenes paired with a caption of a few words that resolved the ambiguity in a positive way (Holmes, Mathews, et al., 2008; Pictet et al., 2011). Participants were instructed to generate a mental image combining the picture and the words.

Each of the 12 sessions started with reminder instructions and a practice example followed by 64 training stimuli arranged into eight sets of 8 with a self-paced break in between each set (cf. Blackwell & Holmes, 2010; Lang et al., 2012). After each stimulus, participants were asked, “How vividly could you imagine the scenario described?” Responses were made on a scale from 1 (*not at all vivid*) to 5 (*extremely vivid*). No individual training stimulus was repeated, so that during the course of the study (12 sessions at home plus the practice session), participants were presented with 416 unique auditory stimuli and 416 unique picture-word stimuli. Participants were scheduled to complete a session of the CBM every day during the 1st week, starting with an auditory session and then alternating between this and the picture-word paradigm, and then 2 sessions in each of the following 3 weeks (1 session of each paradigm). The scheduled order of sessions was the same for all participants. Participants were able to exit a session and resume it at a later time if necessary. If a participant missed completing a session on the scheduled date, the session remained available to complete on a later date.

The intervention in the control condition was identical in all but the following aspects. First, we aimed to remove the training contingency between ambiguity and positive resolution (following Lang et al., 2012). To this end, half of the auditory training scenarios resolved positively and half resolved negatively. Similarly, half of the pictures had positive captions and half had negative captions. Second, we aimed to remove the mental imagery component of the training. To this end, in the auditory paradigm, participants were asked to “focus on the words and meanings” of the training scenarios and after each scenario were asked, “How difficult was it to understand the meaning of the description?” In the picture-word paradigm, participants were instructed to generate a sentence combining the picture and word, and after each picture-word combination they were asked, “How difficult was it to make a sentence combining the picture with the words?” For both paradigms, responses were made on a scale from 1 (*not at all difficult*) to 5 (*extremely difficult*).

The computer system for delivering the interventions via the Internet was built using model-driven tools developed at the University of Oxford’s Department of Computer Science (Davies, Gibbons, Welch, & Crichton, 2014). Participants accessed the intervention from the study Web site using their Web browser, and the HTML interfaces were implemented using Java Server Pages and JavaScript technology. The system was deployed on a secure Web server located in a server room with restricted physical access. Login to the server itself was only through secure channels to a small number of known system administrators. Participants accessed the Web site through an encrypted hypertext transfer protocol secure (https) protocol.

### Measures

#### Clinical history and baseline characteristics

Clinical information, such as anxiety comorbidities and self-reported number of previous episodes of depression, was collected during the SCID interview. Baseline measures included everyday use of imagery (Spontaneous Use of Imagery Scale, SUIS; Reisberg, Pearson, & Kosslyn, 2003), trait anxiety (Trait scale from the State-Trait Anxiety Inventory; Spielberger, Gorsuch, Lushene, Vagg, & Jacobs, 1983), and quality of life (EuroQol-5D-3L, EQ5D; Kind, 1996).

#### Primary outcome of the RCT

The primary outcome measure, as specified in the trial registration, was change in BDI-II score during the 4 weeks from baseline to posttreatment assessment. The BDI-II is a widely used measure of depressive symptoms with scores classified as follows: 0 to 13, minimal depression; 14 to 19, mild depression; 20 to 28, moderate depression; 29 to 63, severe depression. The BDI-II has good psychometric properties whether administered on paper or online (Holländare, Andersson, & Engström, 2010). The anhedonia items on the BDI-II (Item 4: loss of pleasure; Item 12: loss of interest) were summed as in Davidson et al. (2010).

#### Process and mechanisms measures: cognitive targets, in-session vividness/difficulty ratings, and fidelity ratings

Negative interpretive bias was assessed via the Scrambled Sentences Test (SST; Rude et al., 2002) administered under cognitive load (remembering a six-digit number). Participants unscrambled a list of 20 scrambled sentences (e.g., winner born I am loser a) with a time limit of 4 min (as in Blackwell & Holmes, 2010; Lang et al., 2012). A “negativity” score was generated by calculating the proportion of sentences completed correctly with a negative emotional valence (e.g., I am a born loser). Two sets of sentences (baseline and posttreatment) were counterbalanced.

Vividness of positive future imagery was measured via the Prospective Imagery Test (PIT; Stöber, 2000). Participants generated a mental image of 10 positive and 10 negative possible future scenarios and rated each on a 5-point scale for vividness (responses ranged from 1, *no image at all*, to 5, *very vivid*), perceived likelihood of the event happening in the near future (responses ranged from 1, *not at all likely to occur*, to 5, *extremely likely to occur*), and sense of “preexperiencing” of the event (responses ranged from 1, *not at all*, to 5, *completely*; cf. Blackwell et al., 2013).

As described earlier, after each of the training stimuli presented during the Internet intervention, participants gave a rating of vividness (imagery condition) or difficulty (control condition). During each session, the ratings were saved to the server at the end of each block of eight scenarios. For each participant, a grand mean vividness/difficulty rating for the 12-session intervention (i.e., 768 training stimuli) was calculated by averaging the available block means.

At posttreatment, participants completed ratings of their engagement with the intervention during the 4 weeks, which provided a measure of the fidelity with which they had adhered to the experimental manipulation (imagery or verbal processing). These included ratings of use of imagery—“How much did you find yourself thinking in images (i.e., in mental pictures and sensory impressions) as you were listening to the scenarios?” and “How much did you find yourself thinking in images (i.e., in mental pictures and sensory impressions) about the picture-word combinations?”—and use of verbal processing, “How much did you find yourself verbally analysing the meaning of the scenarios as you were listening to them?” and “How much did you find yourself thinking verbally (i.e., in words and making sentences) about the picture-word combinations?” All responses were made on a scale from 1 (*not at all*) to 9 (*all of the time*).

#### Assessment of expectancy and satisfaction

Expectancy was measured at baseline to demonstrate equivalence across conditions. The three expectancy questions from the Credibility and Expectancy Questionnaire (Devilly & Borkovec, 2000) were adapted for the current study. An expectancy score is derived by first standardizing the three individual item scores (across the whole sample) and then summing these three standardized scores (Devilly & Borkovec, 2000).

During the telephone feedback interview at 6-month follow-up, participants gave numerical ratings to the following three questions about their experience of the online intervention: “If you had been offered this online program as a treatment option by your GP or another health professional, how satisfied would you be with what you received?” (rated from 1, *extremely dissatisfied*, to 7, *extremely satisfied*); “How confident would you be about recommending this program to a friend with depression?” (rated from 1, *extremely unconfident*, to 7, *extremely confident*); and “If you were feeling depressed or down in the future, would you be willing to try this program again?” (rated from 1, *extremely unlikely*, to 7, *extremely likely would try again*).

### Procedure

At the baseline assessment, after completion of baseline questionnaire measures and random allocation to condition (for details, see the Randomization section in the appendix), participants completed the SST. A researcher gave a brief overview of the participant’s allocated intervention, after which the participant completed the Expectancy Questionnaire. The researcher then administered a standardized brief (approximately 15-min) introduction to the intervention. In the imagery condition, this included an introduction to mental imagery and practice in generating mental imagery, as in previous studies (e.g., Holmes et al., 2006). The introduction for the control condition was matched in structure and included an introduction to verbal processing and practice in verbal processing adapted from previous experimental studies (e.g., Holmes et al., 2006). Participants then completed a practice session of their allocated intervention, with guidance from the researcher. This consisted of four sets of auditory stimuli and four sets of picture-word stimuli. To increase adherence, at the end of this session, the researcher helped participants to plan when they would complete intervention sessions during the next 4 weeks and explained that completing the sessions in the way instructed may help to improve their mood (following Blackwell & Holmes, 2010; Lang et al., 2012).

A researcher monitored the participant during the subsequent 4 weeks while he/she completed the online intervention from home and sent e-mails to remind the participant about each upcoming session and thank them for each session completed. If a participant missed sessions and did not respond to e-mail contact, phone contact was attempted to promote adherence. Researchers followed a written protocol containing e-mail templates to standardize this contact across participants. A written log of all e-mails, phone calls, and voice mail messages was kept to verify equivalent contact across conditions.

Participants attended a posttreatment assessment approximately 4 weeks after the baseline assessment. After completion of outcome measures, which were administered by a researcher blind to participant allocation (for details, see the Blinding section in the appendix), participants completed the fidelity ratings and a feedback interview. If participants became unable to attend the posttreatment assessment, questionnaire outcome measures were completed online (*n* = 8) or by mail (*n* = 7).

At 1, 3, and 6 months after the end of the 4-week intervention, participants completed follow-up questionnaires online. After participants completed the 6-month questionnaires, they were contacted by phone, and a final feedback interview and debriefing were conducted. If participants did not complete the online questionnaires after receiving reminders, they were mailed a paper copy of the BDI-II with a prepaid return envelope to maximize return of the primary outcome measure.

Participants received reimbursement for their time of 30 pounds ($45) after the posttreatment assessment and a further 10 pounds ($15) on completion of the 6-month follow-up questionnaires. Participants could additionally be reimbursed travel costs for attending the face-to-face assessment sessions.

### Statistical analysis

A sample-size calculation (G*Power 3.1.7; Faul, Erdfelder, Lang, & Buchner, 2007) showed that 128 participants would be needed to provide 80% power to detect a difference of 0.5 (medium effect size *d*) between the two groups on the primary outcome at a 5% significance level, two-tailed. We used 0.5 as a conservative estimate of effect size over 4 weeks following an intention-to-treat analysis of the data from Lang et al. (2012), which obtained a between-groups effect size of 0.67 for the BDI-II for change from baseline to follow-up during a period of 3 weeks. We aimed to recruit 150 participants in total to allow for up to 15% attrition at the primary end point (posttreatment). The main efficacy analyses were carried out in SPSS Version 21 by a statistician (P.W.) not involved in data collection and blind to participant allocation and, according to a predefined analysis plan reviewed by a second statistician, not otherwise involved in the trial. Further analyses were carried out by P.W. and S.E.B. using SPSS Version 22.

#### Main efficacy analyses

Primary comparative analyses between the two groups were conducted by intention to treat, performed as mixed-model repeated measures analysis of variance (ANOVA) with time as within-groups factor and condition as between-groups factor, using the SPSS MIXED command. Mixed-model repeated measures uses all available data with no imputation of missing values, which are assumed to be missing at random, and it is able to fit a general correlation structure between the time points (i.e., no sphericity assumption). Mixed models have been recommended for analyses of trial data (Gueorguieva & Krystal, 2004). A two-level multilevel model was fitted, with an unstructured covariance matrix (Heck, Thomas, & Tabata, 2010). The stratification variables were included as covariates. Estimated mean changes within each group over time, planned contrasts, effect sizes (expressed as Cohen’s *d*), and confidence intervals (CIs) were derived from the mixed-modeling analysis.^2^ Treatment groups were tested at the two-sided 5% significance level. The mixed model was fitted over all five assessment time points and, thus, the comparison between conditions of change from baseline to posttreatment was given by the estimate for this fixed effect (expressed as a *t* statistic) from the mixed model (i.e., as opposed to conducting separate mixed-models analyses to test hypotheses about change from baseline to posttreatment and from baseline to follow-up). The Time × Condition interaction over all five time points therefore gave an estimate of the difference between the patterns of change during the 7 months of assessment between the two conditions and, thus, additionally indicated potential maintenance of benefits during the 6-month follow-up period.

Secondary analyses were also conducted on a “per-protocol” population described in the prespecified statistical analysis plan as those participants judged to have received an adequate “dose” of the intervention, which was defined as more than six sessions completed (i.e., the dose investigated in previous studies; Blackwell & Holmes, 2010; Lang et al., 2012), and who had completed the outcome data within sufficient time of the scheduled date of the assessment to meaningfully relate to that time point: within 2 weeks for the posttreatment assessment and 1-month assessment; within 1 month for the 3-month assessment; and within 2 months for the 6-month assessment. The per-protocol analyses were carried out as for the primary efficacy analyses using mixed-model ANOVAs.

We used a stringent “recovery” criterion to calculate proportions of individuals in each condition who demonstrated “clinically significant change” from baseline on the BDI-II at each time point. Clinically significant change was defined as a reduction in score greater than a reliable change index of 6.90 (Jacobson & Truax, 1991), calculated using the pretreatment standard deviation in our sample and the test-retest reliability from standardization data (*r* = .93), and meeting criteria for recovery (BDI-II score less than 14; Beck et al., 1996). Participants with missing data at the relevant time point in the intention-to-treat sample were classed as “not recovered.”

#### Further process and mechanisms analyses

We carried out analyses of potential subgroup or moderating variables within the same analytic framework as for our main efficacy analyses (i.e., mixed-model repeated measures ANOVAs with time as within-groups factor and condition as between-groups factor, using the SPSS MIXED command and including the stratifying variables as covariates). A subgroup/moderating variable of interest was included in the mixed model as a main effect, two-way interaction terms of Moderator × Condition and Moderator × Time, and a three-way interaction term of Moderator × Condition × Time. A potential moderating effect is indicated by the three-way interaction between the variable of interest, condition, and time (Sun et al., 2012). Where appropriate, moderating effects were investigated within condition via the same mixed model but with the condition term and its interactions omitted.

## Results

**Fig. 1.**
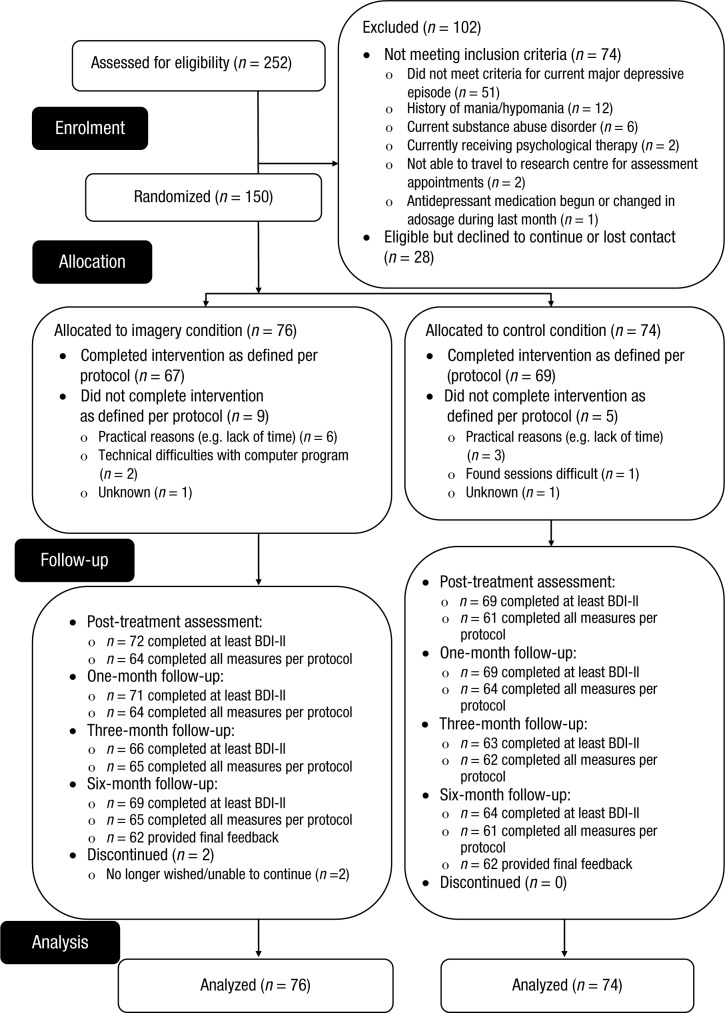
Flow of participants through the trial. BDI-II = Beck Depression Inventory–II.

Participants were recruited from February 2012 to February 2013. Follow-up was completed by November 2013. Recruitment stopped at 150 participants, which was the planned target. Of 252 people assessed for eligibility, 74 did not meet the inclusion criteria, 28 met inclusion criteria but dropped out prior to randomization, and 150 were randomized (see Fig. 1). Overall, 141 (94%) participants completed at least the BDI-II postintervention (primary outcome), and 140 (93%), 129 (86%), and 133 (89%) completed at least this outcome measure at 1-, 3-, and 6-month follow-up, respectively. Attrition was comparable between the two conditions (see Fig. 1), as were baseline characteristics (see Table 1). Participants in the control condition scored significantly higher at baseline than did those in the imagery condition on vividness of negative future imagery (PIT negative vividness), *t*(148) = 2.58, *p* = .011, *d* = 0.42.

**Table 1.** Characteristics of Participants Allocated to Either the Imagery Cognitive Bias Modification or the Control Condition

Characteristic	Imagery (*n* = 76)	Control (*n* = 74)
Women^a^	52 (68%)	51 (69%)
Age (years)	37.64 (14.10)	33.28 (13.73)
BDI-II score by category^a^		
0–28	35 (46%)	32 (43%)
≥ 29	41 (54%)	42 (57%)
White	73 (96%)	69 (93%)
Marital status		
Married or cohabiting	33 (43%)	23 (31%)
Single	30 (40%)	42 (57%)
Separated, divorced, or widowed	13 (17%)	9 (12%)
Employment status		
In paid employment (full-time or part-time)	46 (61%)	39 (53%)
Student	18 (24%)	24 (32%)
Not in employment	12 (16%)	11 (15%)
Housing status		
Home owner	28 (37%)	22 (30%)
Tenant	29 (38%)	30 (41%)
University accommodation	13 (17%)	13 (18%)
Other^b^	6 (8%)	9 (12%)
Years of education		
≤ 11	5 (7%)	6 (8%)
12–15	24 (32%)	19 (26%)
≥ 16	47 (62%)	49 (66%)
Hours of Internet use per week	20.74 (13.76)	21.30 (12.04)
Duration of current episode of depression (months)		
≤ 3	39 (51%)	39 (53%)
4–6	12 (16%)	11 (15%)
7–12	9 (12%)	11 (15%)
> 12	16 (21%)	13 (18%)
Number of previous episodes of depression		
0–1	19 (25%)	18 (24%)
2–3	20 (26%)	13 (18%)
≥ 4	37 (49%)	43 (58%)
Age of onset of depression	21.36 (10.20)	20.31 (10.83)
Current comorbid anxiety disorder	42 (55%)	40 (54%)
Current treatment with antidepressants	33 (43%)	31 (42%)
Ever treated with antidepressants	53 (70%)	52 (70%)
Reported history of psychological therapy/counselling	51 (67%)	41 (55%)
Ever spoken to a health professional about mood	71 (93%)	62 (84%)
Currently have contact with a health professional about mood	23 (30%)	25 (34%)
How heard about study^c^		
Poster advert	5 (7%)	11 (15%)
Radio/newspaper advertisement	22 (29%)	17 (23%)
Internet	32 (43%)	28 (38%)
Other^d^	16 (21%)	18 (24%)
Baseline scores		
BDI-II	29.96 (8.63)	31.14 (10.17)
STAIT	61.00 (6.33)	61.59 (6.87)
EQ5D	61.38 (20.17)	58.88 (18.91)
SUIS	38.50 (9.90)	40.34 (7.91)
PIT		
Positive vividness	2.82 (0.88)	2.87 (0.81)
Positive likelihood	2.54 (0.64)	2.52 (0.66)
Positive experiencing	2.62 (0.81)	2.46 (0.79)
Negative vividness	3.16 (0.89)	3.52 (0.80)
Negative likelihood	3.14 (0.64)	3.30 (0.62)
Negative experiencing	3.09 (0.88)	3.13 (0.74)
SST negativity	0.57 (0.23)	0.60 (0.24)
Anhedonia	3.18 (1.36)	3.47 (1.36)
Expectancy Questionnaire	0.01 (2.59)	–0.01 (2.91)

Note: The table presents number (percentage) or mean (standard deviation) for each measure. BDI-II = Beck Depression Inventory–II (Beck et al., 1996); STAIT = Trait scale of State-Trait Anxiety Inventory (Spielberger et al., 1983); SUIS = Spontaneous Use of Imagery Scale (Reisberg et al., 2003); EQ5D = EuroQol-5D-3L (Kind, 1996); PIT = Prospective Imagery Test (Stöber, 2000); SST = Scrambled Sentences Test (Rude et al., 2002); Anhedonia = Anhedonia items from BDI-II (note *n* = 73 in control condition).

a Stratification variables.

b Living with friends or relatives, or other.

c *n* = 75 in the imagery condition.

d *Other* refers to via university e-mail list, from a friend, unsure, other.

### Adherence and fidelity

Adherence rates to the online program were good and comparable across conditions; 67 participants (88%) completed more than 6 of the 12 intervention sessions (i.e., completed the intervention per protocol) in the imagery condition versus 69 (93%) in the control condition. There was no difference between the two conditions in number of sessions completed (*M*_imagery_ = 10.37, *SD* = 2.95; *M*_control_ = 11.00, *SD* = 2.29), *t*(148) = 1.46, *p* = .15. Participants in the imagery condition reported using imagery significantly more in the intervention sessions than did participants in the control condition (*M*_imagery_ = 7.17, *SD* = 1.04; *M*_control_ = 4.14, *SD* = 1.70), *t*(131) = 12.45, *p* < .001, *d* = 2.16. Participants in the imagery condition reported using verbal analysis significantly less in the intervention sessions than did participants in the control condition (*M*_imagery_ = 3.38, *SD* = 1.59; *M*_control_ = 6.43, *SD* = 1.73), *t*(131) = 10.58, *p* < .001, *d* = 1.84. While completing the intervention, participants in the imagery and control condition received a comparable number of e-mails, phone calls, and voicemails from the researchers (*p*s > .12; see the Participant Contact During the Intervention section in the appendix for details).

### Main efficacy analyses

#### Intention to treat

**Fig. 2.**
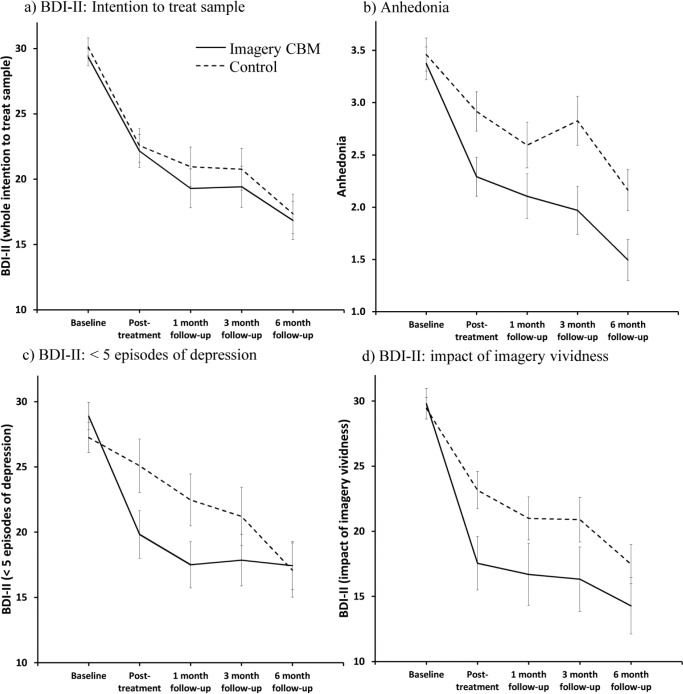
Results: Graphs show (a) primary efficacy analyses (BDI-II, intention to treat), (b) analysis of anhedonia, (c) change in BDI-II in the subgroup of participants with fewer than five depressive episodes, and (d) illustration of change in BDI-II in participants who scored above a “vividness threshold” in the imagery condition (*n* = 27) with the control condition as comparison. Estimated marginal means from the mixed-model analysis are displayed. Error bars represent 1 *SE*. BDI-II = Beck Depression Inventory–II; CBM = cognitive bias modification.

There was no significant difference between the two conditions in change in BDI-II scores from baseline to posttreatment (our primary outcome), *t*(141.11) = 0.21, *p* = .83, *d* = 0.04, 95% CI = [–0.29, 0.36] (see Fig. 2). For the overall mixed model (over five time points), there was a main effect of time, *F*(4, 132.34) = 37.55, *p* < .001, which indicated a decrease in BDI-II scores over the five time points, and no significant effect of condition, *F*(1, 142.76) < 1. The overall interaction of condition and time was nonsignificant, *F*(4, 132.34) < 1. Within-groups effect sizes for change from baseline (*d*) ranged from 0.83, 95% CI = [0.54, 1.13], at posttreatment to 1.45 [1.07, 1.83] at 6-month follow-up in the imagery condition and from 0.74 [0.46, 1.03] at posttreatment to 1.26 [0.89, 1.62] at 6-month follow-up in the control condition. Between-groups effect sizes for change from baseline (*d*) during the follow-up period ranged from 0.07 [–0.25, 0.40] at 1-month follow-up to 0.02 [–0.31, 0.35] at 6-month follow-up. Table S1 in the Supplemental Material available online provides estimated marginal means and effect sizes derived from the mixed-model analysis of BDI-II over the five study time points.

The analyses of our cognitive targets (SST, PIT positive vividness) showed a similar pattern (see Table S2 in the Supplemental Material). For the SST (baseline and posttreatment only), there was a main effect of time, *F*(1, 133.61) = 34.16, *p* < .001, thereby indicating a decrease in scores from baseline to posttreatment, but not condition, *F*(1, 145.13) < 1, and there was no Time × Condition interaction, *F*(1, 133.49) < 1, *d* = 0.05, 95% CI = [–0.29, 0.39]. For PIT positive vividness, there was a nonsignificant trend for a greater increase in the imagery condition compared with the control condition from baseline to posttreatment, *t*(142.57) = 1.86, *p* = .065, *d* = 0.31, 95% CI = [–0.02, 0.64]. In the overall mixed model over all 5 time points, there was a main effect of time, *F*(4, 130.90) = 6.81, *p* < .001, which indicated an increase in vividness scores over time, and no main effect of condition, *F*(1, 143.72) = 1.46, *p* = .23. The overall interaction of condition and time was nonsignificant, *F*(4, 130.90) = 1.28, *p* = .28.

#### Per protocol

The analysis in the per-protocol sample (*n* = 115) yielded a similar pattern of results. There was no significant difference between the two conditions in change in BDI-II from baseline to posttreatment, *t*(113) = 1.02, *p* = .31, *d* = 0.19, 95% CI = [–0.17, 0.56]. In the overall model over five time points, there was a main effect of time, *F*(4, 113) = 34.05, *p* < .001, thereby indicating a decrease in BDI-II scores over time, and no main effect of condition, *F*(1, 111.66) = 1.20, *p* = .28. The overall interaction of condition and time was nonsignificant, *F*(4, 113) < 1 (see Table S1 in the Supplemental Material). Within-groups effect sizes for change from baseline (*d*) ranged from 0.98, 95% CI = [0.62, 1.34], at posttreatment to 1.58 [1.13, 2.03] at 6-month follow-up in the imagery condition and from 0.65 [0.34, 0.95] at posttreatment to 1.23 [0.83, 1.63] at 6-month follow-up in the control condition. Between-groups effect sizes for change from baseline (*d*) during the follow-up period ranged from 0.20 [–0.17, 0.56] at 1-month follow-up to 0.10 [–0.26, 0.47] at 6-month follow-up.

There was no effect of condition on the cognitive targets (see Table S2 in the Supplemental Material). For the SST (*n* = 125), there was a main effect of time, *F*(1, 123) = 30.13, *p* < .001, which indicated a decrease in scores from baseline to posttreatment, but not condition, *F*(1, 121) < 1, and there was no significant Time × Condition interaction, *F*(1, 123) < 1, *d* = 0.02, 95% CI = [–0.33, 0.38]. For PIT positive vividness (*n* = 115), there was no significant difference in change from baseline to posttreatment between the two conditions, *t*(113) = 1.49, *p* = .14, *d* = 0.28. In the overall mixed model, there was a main effect of time, *F*(4, 113) = 5.29, *p* = .001, thereby indicating an increase in vividness scores over time, and no main effect of condition, *F*(1, 110.97) = 1.50, *p* = .22. The overall interaction of condition and time was nonsignificant, *F*(4, 113) < 1.

#### Clinically significant change

For the intention-to-treat sample, 17.1%, 95% CI = [10.2, 27.3], of participants in the imagery condition and 21.6% [13.7, 32.4] in the control condition demonstrated clinically significant change (as defined by our recovery criteria) at posttreatment. By 6-month follow-up, these figures were 36.8% [26.9, 48.1] and 31.1% [21.7, 42.4], respectively. For the per-protocol sample, 18.6% [10.6, 30.6] of participants in the imagery condition and 21.4% [12.6, 34.0] of those in the control condition demonstrated clinically significant change at posttreatment. By 6-month follow-up, these figures were 40.7% [29.1, 53.5] and 35.7% [24.5, 48.9], respectively.

#### Satisfaction ratings

Participant ratings at 6-month follow-up were significantly higher in the imagery condition than in the control condition—satisfaction: *M*_imagery_ = 4.79, *SD* = 1.32 vs. *M*_control_ = 3.89, *SD* = 1.57, *t*(122) = 3.47, *p* = .001, *d* = 0.62; confidence in recommending to a friend: *M*_imagery_ = 5.24, *SD* = 1.31 vs. *M*_control_ = 3.84, *SD* = 1.79, *t*(122) = 4.97, *p* < .001, *d* = 0.89; willingness to try again: *M*_imagery_ = 5.55, *SD* = 1.72 vs. *M*_control_ = 4.55, *SD* = 2.16, *t*(122) = 2.85, *p* = .005, *d* = 0.51.

### Why did we not find the expected superiority of the imagery condition in our main efficacy analyses?

After the unexpected finding that there was no difference in outcomes between the imagery and control conditions in our main efficacy analyses, we carried out further exploratory analyses to test subsequent hypotheses about why we might not have obtained the expected results. Given that these hypotheses were concerned with mechanisms by which the interventions may or may not have led to change rather than broader efficacy questions, we used the per-protocol sample for these analyses unless otherwise specified.

#### Did the imagery condition have a specific effect on anhedonia?

Although the imagery CBM did not lead to greater improvement than the control condition on the whole constellation of depressive symptoms (as measured by the BDI-II, which includes cognitive, affective, and somatic symptoms), it may have a more specific effect on aspects of depressive symptoms that were more closely matched to what was practiced in the training itself, that is, imagining oneself enjoying or taking interest in activities. We therefore investigated whether imagery CBM could have led to increased enjoyment or interest in activities in day-to-day life, that is, decreased anhedonia. We found that there was a significantly greater reduction in anhedonia in participants in the imagery condition compared with the control condition from baseline to posttreatment, *t*(113.18) = 2.17, *p* = .032, *d* = 0.41, 95% CI = [0.04, 0.78], although this was not consistently maintained over all follow-up time points, as indicated by a nonsignificant Time × Condition interaction in the overall mixed model fitted over all five time points, *F*(4, 112.42) = 1.67, *p* = .16 (see Fig. 2). The mixed model showed a main effect of time, *F*(4, 112.42) = 26.64, *p* < .001, which indicated a decrease in anhedonia over the study time points, and a main effect of condition, *F*(1, 111.48) = 6.67, *p* = .011, thereby indicating that on average across the five time points, anhedonia scores were lower in the imagery condition compared with the control condition (see Table S1 in the Supplemental Material).

#### Did the imagery condition only lead to superior outcomes in a subgroup of our clinically heterogeneous sample?

We next investigated the possibility that imagery CBM exerted a beneficial effect in specific subgroups of the broad range of depressed patients recruited to our trial. The subgroups examined were guided by the CBM and depression treatment literature and defined using both a measure of illness recurrence (number of episodes of depression) and the stratification variables employed in the study (gender, baseline depression severity). Evidence for moderation was indicated by a significant three-way interaction (Time × Condition × Subgroup) in the mixed model (with BDI-II as dependent measure).

In terms of illness recurrence, our sample was characterized by a high number of episodes of depression. We found a significant moderating effect of number of episodes of depression, *F*(4, 111) = 2.99, *p* = .022, with our sample split at the midpoint into participants with fewer than five (*n* = 55) versus those with five or more (*n* = 60) depressive episodes. This corresponds to the cutoff in the trial by Bockting et al. (2005), whose sample had similar levels of recurrence and medication use to ours. Within the subgroup with fewer than five depressive episodes, there was a significantly greater reduction in BDI-II from baseline to posttreatment in the imagery condition compared with the control condition, *t*(53) = 2.66, *p* = .01, *d* = 0.73, 95% CI = [0.19, 1.28]. For the overall mixed model, the Time × Condition interaction was significant, *F*(4, 53) = 2.60, *p* = .047 (see Fig. 2). Investigation of this interaction across the assessment time points revealed that the reduction in symptoms of depression from baseline was significantly greater in participants in the imagery condition compared with the control condition at 1-month follow-up, *t*(53) = 2.66, *p* = .01, *d* = 0.73, 95% CI = [0.18, 1.28], but no longer at 3-month follow-up, *t*(53) = 1.70, *p* = .10, *d* = 0.47, 95% CI = [–0.07, 1.00] (see Table S1 in the Supplemental Material). The mixed model also showed a main effect of time, *F*(4, 53) = 15.51, *p* < .001, but not of condition, *F*(1, 53) = 1.35, *p* = .25. Within the subgroup with five or more depressive episodes, there was no difference between the conditions in reduction in BDI-II from baseline to posttreatment, *t*(58) < 1, and no Time × Condition interaction in the overall mixed model, *F*(4, 58) = 1.06, *p* = .39. There was a main effect of time, *F*(4, 58) = 20.68, *p* < .001, but not of condition, *F*(1, 57.67) < 1.

We found no evidence of moderation for our stratification variables (baseline BDI-II category, gender; cf. Blackwell & Holmes, 2010; Lang et al., 2012) or for age (cf. Blackwell & Holmes, 2010), current antidepressant use (e.g., Browning et al., 2011), baseline bias (SST; cf. Micco, Henin, & Hirschfeld-Becker, 2014), or questionnaire-measured imagery ability (PIT positive vividness, SUIS; cf. Lang et al., 2012).

#### Was the imagery intervention helpful only for those participants who successfully engaged with imagery?

We next investigated whether engaging with a putative active component of the imagery CBM—vividly imagining the training scenarios—was necessary for participants to benefit from the imagery condition.

Within the imagery condition, we carried out the same mixed-model analysis as for the main efficacy analyses, with BDI-II as outcome, but included the mean vividness rating (*M* = 3.42, *SD* = 0.71) as a covariate. A significant Time × Vividness interaction, *F*(4, 57) = 3.66, *p* = .010, indicated that the more vividly participants in the imagery condition imagined the training scenarios during the 4-week intervention, the greater their reduction in symptoms of depression during the course of the study.

In fact, the same moderating effect of imagery was found when vividness ratings from participants’ very first attempt at the task (first block of the practice session, i.e., eight scenarios; *M* = 3.59, *SD* = 0.71) were considered—Interaction × Time: *F*(4, 62.17) = 2.98, *p* = .026—using all participants in the imagery condition for whom these data were available (*n =* 72; the data did not save correctly for 4 participants). This suggests that it may be possible to estimate the likely impact of the imagery training on participants’ depression on the basis of only an initial eight-scenario assessment of the ease with which they engage in the task and before these ratings could be influenced by improvement in depression.

Within the control condition, there was no evidence for a relationship between difficulty ratings and reduction in BDI-II, as indicated by a nonsignificant Time × Difficulty interaction, *F*(4, 54) < 1, in an equivalent mixed-model analysis that included the mean difficulty rating (*M* = 1.55, *SD* = 0.60) as a covariate.

We investigated what level of imagery vividness was required for participants in the imagery condition, compared with those in the control condition, to experience a greater reduction in symptoms of depression from baseline to posttreatment. A regression within the imagery condition (dependent variable: change in BDI-II from baseline to posttreatment; independent variable: mean vividness score) indicated that a mean vividness of more than 3.52 (where 3 = *somewhat vivid* and 4 = *very vivid*) was required in order for the reduction in BDI-II to be greater than the upper bound (95% CI) of the mean decrease within the control condition of 8.84, corresponding to 27 participants or 45.8% of our per-protocol sample. This result is illustrated in Figure 2c, which plots the change in score on the BDI-II in the subgroup of participants in the imagery condition who reached this “vividness threshold” against the change in the control condition for comparison (estimated marginal means are derived from a mixed model analysis using this subsample).

#### Was the reduction in symptoms of depression related to changes in the cognitive targets?

Given that there was no significant difference between conditions in change in cognitive targets from baseline to posttreatment, as assessed by either SST negativity or PIT positive vividness, we collapsed scores across both conditions to investigate the relationship between cognitive targets and symptoms of depression.^3^ At baseline, BDI-II correlated significantly with SST negativity, *r*(115) = .51, *p* < .001, but there was a nonsignificant trend with PIT positive vividness, *r*(115) = –.16, *p* = .09. Reduction in BDI-II from baseline to posttreatment correlated significantly with both reduction in SST negativity, *r*(113) = .46, *p* < .001, and increase in PIT positive vividness, *r*(115) = .39, *p* < .001. We carried out a regression with baseline BDI-II and change in both SST negativity and PIT positive vividness from baseline to posttreatment as predictors and change in BDI-II from baseline to posttreatment as dependent variables. Change in both SST negativity (β = 0.35, *p* < .001) and PIT positive vividness (β = 0.28, *p* = .001) independently predicted reduction in BDI-II. We investigated whether bias at posttreatment was predictive of future depression. When we controlled for BDI-II posttreatment, SST negativity posttreatment was not correlated with BDI-II at 1-month follow-up, *r*(110) = .14, *p* = .15, but was correlated significantly with BDI-II at 3-month follow-up, *r*(110) = .22, *p* = .019, and at 6-month follow-up, *r*(110) = .22, *p* = .02. PIT positive vividness at posttreatment was not correlated significantly with BDI-II at any follow-up time point.

## Discussion

We carried out an RCT in which 150 participants with current major depression were assigned to a 4-week imagery CBM or an active control condition, delivered via the Internet, and assessed up to 6-months posttreatment. There was a high completion rate for the Internet-delivered CBM, and participant feedback was positive. In our planned analyses, we did not find the expected superiority of the imagery CBM over the control condition in reducing symptoms of depression (our primary outcome measure), reducing negative interpretive bias, or increasing vividness of positive future imagery. Participants in both conditions showed equal improvements on all these measures.

However, exploratory post hoc analyses revealed that participants in the imagery CBM condition experienced a greater improvement in anhedonia (a subgroup of depressive symptoms related to the loss of interest in or enjoyment from activities) over the intervention compared with those in the control condition. Furthermore, within the subgroup of participants with fewer than five episodes of depression, there was a significantly greater reduction in symptoms of depression as a whole in the imagery CBM condition compared with the control condition. Finally, we found that the more vividly participants in the imagery CBM condition reported imagining the training scenarios during the intervention, the more their symptoms of depression reduced. Participants in the imagery CBM condition who engaged to a threshold level of imagery vividness experienced a greater reduction in symptoms of depression as a whole than did participants in the control condition. Vividness ratings in just the initial practice session also predicted subsequent reduction in symptoms of depression. These results from the trial highlight potentially fruitful pathways for further development of imagery CBM as a low-intensity treatment tool in the context of depression.

In reflecting on the results of the current study, it is worth noting some points of RCT methodology, as the majority of CBM research has taken place within the context of experimental studies. The close adherence to a clinical trials framework is a strength of the current study (Kiluk et al., 2011). We had a sufficiently large sample, with good adherence rates and data coverage, to provide robust estimates of effect sizes and also a relatively “real-world” sample, for example, with many previous depressive episodes and concurrent comorbidities. We carried out face-to-face diagnostic assessments with all participants and, thus, have a well-characterized sample, which is often not possible with Internet RCTs (Andersson & Titov, 2014). However, it is also worth noting that in using relatively broad inclusion criteria, rather than the more restrictive criteria often used in efficacy RCTs (e.g., von Wolff et al., 2014), we obtained a comparatively heterogeneous sample, which may have increased the variance and decreased the signal-to-noise ratio in comparison with standard efficacy RCTs. For example, we did not exclude participants from analysis on the basis of depression severity or suicidality.

Prior to the present trial, our experimental studies in nonclinical samples had demonstrated increased positive affect as an outcome of imagery CBM, compared with a control condition (Holmes, Coughtrey, & Connor, 2008; Holmes et al., 2006; Holmes, Lang, & Shah, 2009; Nelis et al., 2012; Pictet et al., 2011; Rohrbacher, Blackwell, Holmes, & Reinecke, 2014). Consistent with these prior results, exploratory post hoc analyses of the present results revealed a significantly greater reduction in anhedonia over the 4-week intervention in the imagery CBM condition compared with the control condition. Anhedonia is a core symptom of depression that is relatively resistant to current first-line therapies but is predictive of poorer treatment outcomes (Treadway & Zald, 2011; Uher et al., 2012) and is recognized as an important but neglected clinical target in its own right (e.g., Insel, 2012). Although, in the current study, we did not find the expected between-groups difference for reduction in symptoms of depression as a whole, the indication that imagery CBM may contribute to reducing anhedonia is worth pursuing, as neither current psychological nor current pharmacological treatments are adequate in this regard.

Our exploratory post hoc finding that the imagery CBM was more effective than the control condition in reducing symptoms of depression among participants with fewer than five episodes of depression (albeit not in the full sample) suggests that the positive imagery aspect of the intervention may be more useful for individuals whose depression is less recurrent. This contrasts with psychological treatment studies that target relapse prevention, which have tended to find greater usefulness for people with more (rather than fewer) episodes of depression (e.g., Bockting et al., 2005; Teasdale et al., 2000). However, this subgroup analysis in our RCT was not specified prior to the trial, and clearly this finding needs replication. A future trial could include number of depressive episodes as a stratification variable with a planned subgroup analysis (Sun et al., 2012). Were a precise number of depressive episodes to be of interest, a structured assessment, such as a timeline (e.g., Crane et al., 2014), may be useful.

The more vividly participants reported imagining the positive training scenarios during the imagery CBM, the more their symptoms of depression were reduced. Vividly imagining the training scenarios is thought to be an active ingredient in the effects of the imagery CBM. Several previous studies (Holmes, Coughtrey, & Connor, 2008; Holmes et al., 2006; Holmes, Lang, & Shah, 2009; Nelis et al., 2012; Torkan et al., 2014) have shown that only when participants were instructed specifically to imagine the positive training scenarios (e.g., as opposed to processing the information verbally) did they show positive changes in mood and cognitive bias. Our current vividness findings suggest that in the measurement of adherence to computerized interventions (cf. Kiluk et al., 2011), it is important to include measures of “active” adherence (i.e., engaging with the intervention; here, vividness of imagery) rather than simply “passive” adherence (e.g., going through the motions of completing sessions; cf. Bendelin et al., 2011, in relation to iCBT).

Vividness ratings made for just the first eight training scenarios in the initial practice session of the imagery CBM were predictive of reduction in symptoms of depression. This makes it unlikely that enhanced vividness was a secondary consequence of symptom improvement rather than being an active agent. If so, it seems important in future research to identify people who may need further training in imagery prior to starting the intervention (cf. Lang et al., 2012; Steel et al., 2010).

The critical question remaining is that of why the main primary outcome analyses of BDI-II scores failed to reveal any differences between groups. It may be that neither the imagery CBM condition nor the control condition had any beneficial effects on depression overall or, alternatively, that both conditions were equally effective. In the absence of a no-intervention (e.g., wait list) control, we have no direct evidence that the change in symptoms of depression was any greater than in the absence of any intervention. In recent meta-analyses, Cuijpers et al. (2012) reported a pre- to posttreatment effect size (*g*) of 0.39, 95% CI = [0.03, 0.74], in waiting-list/care-as-usual groups, and Rutherford, Mori, Sneed, Pimontel, and Roose (2012) reported an effect size (*d*) of 0.51 [0.27, 0.74] over wait lists with a mean duration of 10 weeks. Lack of overlap between these CIs and those for the within-groups effect sizes in our imagery condition (and limited overlap for the control condition) during a similar time period (1-month follow-up: *d*_imagery_ = 1.16, [0.85, 1.48], *d*_control_ = 0.90, [0.61, 1.20]; 3-month follow-up: *d*_imagery_ = 1.15, [0.81, 1.49], *d*_control_ = 0.92, [0.60, 1.24]) suggests that greater change occurred in the current trial than would be expected during the same time with no intervention. The within-groups effect sizes in the current RCT are in the same range as those reported for change in symptoms of depression from pre- to posttreatment in trials of Internet-delivered psychological therapies for depression, with Cohen’s *d*s of 1.35, 0.95, and 0.78 for therapist-supported, administrative-supported, and unsupported therapies, respectively (Richards & Richardson, 2012). However, any inferences from these indirect comparisons can be regarded only as hypotheses for testing in future studies.

Even if it is granted that both groups showed some improvement, it remains unclear why there were no differences in BDI-II change between the imagery CBM condition and the control condition. Our previous experimental studies all found differences between groups assigned to similar interventions and control conditions (Holmes, Coughtrey, & Connor, 2008; Holmes et al., 2006; Holmes, Lang, & Shah, 2009; Nelis et al., 2012; Rohrbacher et al., 2014), including those with clinical samples (Lang et al., 2012; Torkan et al., 2014; Williams et al., 2013). However, these previous studies also showed a differential effect of imagery CBM versus the control condition on cognitive targets (e.g., measures of bias), which we assume is the mechanism driving mood change. In the current trial, we did not find differential change in cognitive targets between conditions; thus, differential effects on symptom outcomes may not be expected (Clarke, Notebaert, & MacLeod, 2014). Symptom change was related to the extent of change in cognitive targets, although measurement of these at only pre- and posttreatment limits attributions of causality (Kraemer, Wilson, Fairburn, & Agras, 2002; Mogoaşe, David, & Koster, 2014). Comparison of the within-groups effect sizes for BDI-II change in the current RCT with those from studies investigating a 1-week imagery CBM intervention (Blackwell & Holmes, 2010; Lang et al., 2012; Torkan et al., 2014; Williams et al., 2013) suggests that we obtained both less improvement than expected in the imagery condition and more improvement than expected in the control condition. There are a number of differences between the previous studies and the current RCT that may account for this unexpected finding.

The current study was conducted within a clinical-trials framework, including prospective registration, independent randomization and robust allocation concealment, intention-to-treat principles for our main analyses, and blinding of outcome assessors (Moher et al., 2010). This was not the case for previous studies in which, for example, the outcome assessors were not blind to participant allocation, thereby potentially allowing for more demand effects. Furthermore, a clinical trial may attract different participants, with different expectancies and experiences, which can have an impact on outcomes and the ability to detect between-groups effects (cf. Rutherford & Roose, 2013). From this perspective, it is possible that we were overoptimistic in our expectation of a medium between-groups effect size and that anticipation of a small between-groups effect size (as we found in the current per-protocol analysis for the BDI-II) would have been more realistic. Given the low-intensity nature of the intervention (the median session duration was just less than 20 min, which means that for most people, the 12-session intervention came to less than 4 hr in total), a small between-groups effect size may in fact still reflect a cost-effective intervention technique and, thus, be worth pursuing. However, the sample size required to have adequate power to detect such an effect (*N* = 788 for 80% power at 5% significance level) was beyond the scope of this initial trial.

The current study also differed from previous research in the duration, schedule, and location of the active intervention. It may be that the more an intervention moves from the confines of tightly controlled laboratory sessions, the more the individual variation in adherence to the intended manipulation and, thus, the smaller any between-groups effects will become. Furthermore, it is possible that most of the within-session learning happens in the early sessions and, thus, an increase in the number of sessions and the length of the schedule (as in the current trial) leads to diminishing returns (cf. Eberl et al., 2014; Stafford & Dewar, 2014).

Conversely, our control condition may have benefitted by the extension of the training schedule. This condition may have contained some of the active components of the intervention, for example, the exposure to ambiguous information (which theoretically may activate the potential competing meanings; cf. Clarke, Nanthakumar, et al., 2014; Mathews, 2012) and the experience of this ambiguity being resolved, both positively and negatively, which may enhance cognitive flexibility (anticipation that either outcome is possible). From the experimental single-session literature, one might expect a control condition with no imagery instructions and no valence-specific training contingency to exert little impact on cognitive bias during a single session and, thus, be considered “inert.” However, factors having no impact within single-session studies, such as reflective processes that take more time to consolidate and exert effects, may gain in importance during longer periods. Clinical translation of CBM could benefit from moving beyond regarding CBM interventions as simply repeated instances of single sessions and considering more broadly the mechanisms by which differential engagement in the intervention could influence clinical outcomes (cf. Blackwell & Holmes, 2010; Standage, Harris, & Fox, 2014).

A major limitation of the current study is the lack of a no-intervention (e.g., wait list) control; thus, we do not have direct evidence that the change observed in our participants was more than would have been observed in the absence of any intervention. However, a broader perspective of the psychological treatment field suggests that simply finding another psychological treatment with superior efficacy to a wait list may not be the optimal solution. Numerous treatments that are better than a wait list (Cuijpers, van Straten, Bohlmeijer, Hollon, & Andersson, 2010) already exist, and it has been argued that psychological treatment development could benefit from moving away from a reliance on wait list controls and instead carrying out more rigorous tests against appropriately matched control conditions (Boot, Simons, Stothart, & Stutts, 2013; Furukawa et al., 2014), as in CBM studies (Clarke, Notebaert, & MacLeod, 2014). However, CBM researchers may benefit from consideration of whether a control condition used in an experimental study to isolate one specific mechanism is always the most useful control condition for a trial aimed at establishing clinical efficacy. The development of control conditions that are similar to the placebos used in pharmacological trials, as in superficially resembling the intervention but containing none of its active ingredients (as may have occurred here), may be a useful way forward.

Our choice of primary outcome—depressive symptoms as measured by the BDI-II—was broad. An intervention may target a specific process very effectively, but if success is evaluated primarily via a broader measure of disorder, this could lead to underestimation of its effectiveness (Reardon, 2014). In the case of positive imagery CBM, the underlying cognitive model (i.e., repeated simulation of positive outcomes; Holmes, Lang, & Deeprose, 2009) and current findings suggest that in future studies, a measure of anhedonia may be worth exploring as a more specific primary outcome measure (rather than depressive symptoms as a whole). It would be useful to include separate measures of anhedonia in addition to that provided by BDI-II (Davidson et al., 2010).

In conclusion, our findings are in one sense negative: They did not support the proposal that positive imagery CBM is an effective treatment for depression. However, there is also some suggestion from exploratory analyses that imagery CBM may have potential as a technique to improve anhedonia or to help individuals with less recurrent depression or those who engage to a threshold level of imagery. We consider each of these possibilities in the following paragraphs. It is worth noting that the field of CBM is young and currently in the early stages of clinical translation (Clarke, Notebaert, & MacLeod, 2014; Fox, Mackintosh, & Holmes, 2014). An unexpected result, as in the current RCT, can be informative in a variety of ways, as we have discussed. For example, there may be wider implications for the field concerning the choice of control condition or of outcome measurement in moving from the lab to the clinic.

In future work, researchers should seek to explore and extend implications of the current RCT. Various methodological options are worthy of consideration. A brief “run-in” phase for adherence to the treatment requirements (i.e., to engage to a threshold level of imagery) might be used prior to randomization—a strategy beginning to be used in some pharmacological RCTs (Geddes et al., 2010). The training itself could also be refined. Specifically, we suggest the need for evaluation of improved versions of imagery CBM suitable for real-world (rather than lab) training environments. For example, participants may be guided to achieve higher standards of vivid positive self-referent imagery prior to entry into the main phase of the intervention and maintain this during the course of the intervention via feedback processes.

Such improved imagery CBM versions would need to be evaluated in comparison with different types of control conditions that are more convincingly inert in the longer term (e.g., by avoiding any emotional material, or without resolving ambiguity either positively or negatively). Nonetheless, the apparent improvement seen in the present control condition raises interesting questions about idiosyncratic ways in which participants may engage with training delivered via the Internet. The common practice of using equal frequencies of negative and positive resolutions of ambiguity as a control condition in CBM may require reconsideration when used in more prolonged interventions, and the potential benefit of inducing a more flexible emotional response style needs to be further evaluated.

The results of the current RCT suggest that the efficacy of imagery CBM within a sample of depressed individuals with few prior episodes of depression should be investigated. In fact, within the general population, a slight majority of patients with major depression may fall below the split point in the current study of five episodes, given that the median number of lifetime depressive episodes is four (Judd, 1997). In a heterogeneous disorder such as depression, identifying particular subgroups of patients for whom an intervention may be particularly helpful, or patient stratification, is an important part of treatment development (Kazdin & Blase, 2011).

The potential for imagery CBM to improve anhedonia, as indicated in the current RCT, should be followed up, given that this core symptom of depression poses a major challenge to current treatments (Insel, 2012; Treadway & Zald, 2011). A useful first step may be to better characterize the effects of imagery CBM by using richer measures of anhedonia that relate to its various facets (e.g., anticipation vs. experience of pleasure; Dunn, 2012; Pizzagalli, 2014). If it is indeed the case that imagery CBM can improve anhedonia, then it may have particular value as an adjunct to current treatment approaches (psychological or pharmacological) that improve other symptoms of depression while leaving anhedonia relatively untouched. An experimental psychopathology approach could be used to investigate whether imagery CBM and treatment approaches such as iCBT or antidepressants do in fact work via complementary mechanisms, given that treatments often do not combine in an additive manner (Browning et al., 2011). Overall, a consideration of outcome measures, alongside refined methodology as discussed earlier, would allow researchers to assess whether the preliminary indications of benefit seen here can be replicated.

## Supplementary Material

Supplementary material

## Appendix

### Trial sites and approvals

The trial was sponsored by the University of Oxford. Research and development approval was obtained from Oxfordshire Primary Care Trust and Oxford Health NHS Foundation Trust to act as participant identification centers. All recruitment and face-to-face assessment procedures took place at the main research site, the Department of Psychiatry, University of Oxford. In April 2013, the MRC Cognition and Brain Sciences Unit, Cambridge, was added as a subsidiary site for remote collection of remaining follow-up data and for conducting data analyses. The relevant management approvals were obtained for both sites.

### Randomization

Randomization took place at the baseline assessment after completion of all baseline questionnaire measures. Participants were randomized in a 1:1 ratio to one of two groups, imagery cognitive bias modification or control, within the constraints of stratification by gender and baseline Beck Depression Inventory–II score (mild-to-moderate score of 28 or less vs. severe score of 29 or more). The researcher who carried out the baseline assessment assigned participants to their allocated intervention via a Web-based randomization system written by the clinical trials software development team in the Oxford Cognitive Health and Neurosciences Clinical Trials Unit and hosted by the University of Oxford Medical Sciences Information Management Unit. Randomization was carried out using a secret allocation table, generated by the University of Oxford Centre for Statistics in Medicine, with a variable-length block structure to ensure that allocations were balanced and could not be predicted by trial staff.

### Blinding

Blinding of participants to allocation was achieved by having only those aspects of the study interventions that were common to both conditions (e.g., schedule of sessions and general nature of tasks, such as listening to scenarios, responding to pictures) in the study information. Participants were debriefed about the nature of the two different study conditions at the end of the trial (once they had completed the 6-month outcome measures) and, thus, did not know the specifics of the other condition until they had finished the trial. In the face-to-face posttreatment assessment, a researcher blind to participant allocation was assigned to administer the outcome questionnaires, and blinding was achieved for at least the primary outcome with one exception (due to an administrative oversight). Thus, the trial can be considered “double blind.”

The trial database was maintained as blind until all data collection, checking, and cleaning for the main efficacy analyses had taken place; after this, participant condition was downloaded from the randomization service and inserted into the database. Initial data checking and cleaning was carried out by S.E.B. and research assistants involved in data collection, and a random selection of 10% of baseline and posttreatment data were then additionally checked by another researcher not otherwise involved in the study and blind to participant condition.

### Participant contact during the intervention

Contact from the researchers during the 4-week intervention included e-mails (*M_imagery_* = 18.95, *SD* = 3.77; *M_control_* = 18.14, *SD* = 3.24), *t*(148) = 1.41, *p* = .16; phone calls (*M_imagery_* = 0.33, *SD* = 0.93; *M_control_* = 0.24, *SD* = 0.57), *t*(148) = 0.68, *p* = .50; and voice mail messages (*M_imagery_* = 0.18, *SD* = 0.58; *M_control_* = 0.07, *SD* = 0.30), *t*(148) = 1.53, *p* = .13.

## Notes

1. That is, corresponding to the primary outcome measure as prespecified in the clinical trials registration. Following recommendations for RCTs (Moher et al., 2010), we prespecified one measure as the primary outcome measure in our trial protocol and registration and powered the RCT accordingly. In taking the step from previous smaller-scale studies to the current RCT, we decided to preserve the same outcome measure (Beck Depression Inventory–II) for which we had found change in these earlier studies.

2. Effect sizes for change from baseline within conditions, expressed as Cohen’s *d*s, were calculated as the mean change from baseline estimated from the mixed model divided by the standard deviation (raw score) at baseline. Effect sizes for the difference in change from baseline between conditions, expressed as Cohen’s *d*s, were calculated using the *t* and degree-of-freedom values for the relevant fixed-effect estimates derived from the mixed model by using the *d* = 2*t*/sqrt(*df*) formula (Rosenthal & Rosnow, 1991). CIs for *d* values were calculated via the formulae provided by Viechtbauer (2007) using estimated parameters from the mixed model. CIs for proportions were calculated via the modified Wald method (Agresti & Coull, 1998).

3. There was no difference between conditions in the relationship between change in these cognitive targets and change in scores on the BDI-II. Two participants within the BDI-II per-protocol sample did not complete the SST at posttreatment.
