# Supplementary material for: Positive Imagery-Based Cognitive Bias Modification as a Web-Based Treatment Tool for Depressed Adults: A Randomized Controlled Trial
Source: Clin Psychol Sci. 2015 Jan;3(1):91–111. doi: 10.1177/2167702614560746 (PMC4359210; doi:10.1177/2167702614560746)
Supplement: Supplementary material [file Holmes_Blackwell_et_al_Supp_Tables.pdf]

Supplementary Materials Available Online

Supplementary Table 1. Results from the mixed model analyses including estimated marginal means and effect sizes for a) BDI-II (primary outcome), b) anhedonia, c) subgroup with fewer than five depressive episodes.

		a) BDI-II in full sample				
		Baseline	Post-treatment	1-month	3-month	6-month
Intention to treat						
Imagery	*M*	29.35	22.16	19.30	19.41	16.84
(*n* =76)	*SD*	8.63	10.86	12.88	13.07	12.25
	d_{within} [95% CIs]		0.83 [0.54, 1.13]	1.16 [0.85, 1.48]	1.15 [0.81, 1.49]	1.45 [1.07, 1.83]
Control	*M*	30.12	22.58	20.95	20.76	17.35
(*n* =74)	*SD*	10.17	11.03	13.02	13.32	12.69
	d_{within} [95% CIs]		0.74 [0.46, 1.03]	0.90 [0.61, 1.20]	0.92 [0.60, 1.24]	1.26 [0.89, 1.62]
Comparison	*t*(df)		0.21 (141.11)	0.45 (143.96)	0.27 (133.72)	0.13 (138.28)
	p		.83	.66	.79	.90
	$d_{between}$ [95% CIs]		-0.04 [-0.36, 0.29]	0.07 [-0.25, 0.40]	0.05 [-0.29, 0.38]	-0.02 [-0.35, 0.31]
Per protocol						
Imagery	*M*	29.17	21.02	18.53	18.83	16.02
(*n* =59)	*SD*	8.33	10.66	12.12	12.85	11.61
	d_{within} [95% CIs]		0.98 [0.62, 1.34]	1.28 [0.88, 1.67]	1.24 [0.85, 1.64]	1.58 [1.13, 2.03]
Control	*M*	29.53	23.25	21.08	20.98	17.57
(*n* =56)	*SD*	9.74	10.74	12.25	13.01	11.72
	d_{within} [95% CIs]		0.65 [0.34, 0.95]	0.87 [0.55, 1.19]	0.88 [0.54, 1.22]	1.23 [0.83, 1.63]
Comparison	*t*(df)		1.02 (113)	1.05 (113)	0.78 (113)	0.54 (113)
	p		.31	.30	.44	.59
	$d_{between}$ [95% CIs]		0.19 [-0.17, 0.56]	0.20 [-0.17, 0.56]	0.15 [-0.22, 0.51]	0.10 [-0.26, 0.47]

		b) Anhedonia				
		Baseline	Post-treatment	1-month	3-month	6-month
Imagery	*M*	3.38	2.29	2.10	1.97	1.49
(*n* =59)	*SD*	1.27	1.46	1.68	1.80	1.53
	d_{within} [95% CIs]		0.85 [0.53, 1.18]	1.00 [0.61, 1.39]	1.11 [0.69, 1.52]	1.48 [1.03, 1.93]
Control	*M*	3.46	2.91	2.59	2.83	2.16
(*n* =56)	*SD*	1.34	1.46	1.69	1.80	1.53
	d_{within} [95% CIs]		0.41 [0.15, 0.67]	0.65 [0.36, 0.93]	0.47 [0.17, 0.77]	0.97 [0.61, 1.33]
Comparison	*t*(df)		2.17 (113.18)	1.32 (110.06)	2.20 (112.56)	1.83 (112.57)
	p		.03	.19	.03	.07
	$d_{between}$ [95% CIs]		0.41 [0.04, 0.78]	0.25 [-0.12, 0.62]	0.42 [0.04, 0.79]	0.34 [-0.02, 0.71]
		c) BDI-II: subgroup with fewer than five episodes of depression				
		Baseline	Post-treatment	1-month	3-month	6-month
Imagery	*M*	28.89	19.82	17.50	17.85	17.44
(*n* =31)	*SD*	8.36	9.80	9.54	10.57	9.88
	d_{within} [95% CIs]		1.08 [0.52, 1.65]	1.36 [0.76, 1.96]	1.32 [0.72, 1.92]	1.37 [0.75, 1.99]
Control	*M*	27.26	25.09	22.47	21.22	17.09
(*n* =24)	*SD*	9.13	10.84	10.55	11.73	10.93
	d_{within} [95% CIs]		0.24 [-0.08, 0.56]	0.52 [0.18, 0.87]	0.66 [0.24, 1.08]	1.11 [0.50, 1.72]
Comparison	*t*(df)		2.66 (53)	2.66 (53)	1.70 (53)	0.42 (53)
	p		.01	.01	.09	.68
	$d_{between}$ [95% CIs]		0.73 [0.19, 1.28]	0.73 [0.18, 1.28]	0.47 [-0.07, 1.00]	0.12 [-0.41, 0.64]

Note. Means, standard deviations, effect sizes and confidence intervals are all derived from the mixed model analysis, with the exception of pre-treatment standard deviation, which is presented as the raw score. d_{within} = within-group effect size for change from baseline to the specified time-point. The *t* and *p* values are for the comparison between conditions of the change from baseline to the specified timepoints (estimates of fixed effects from the mixed model). $d_{between}$ = between-group effect size, comparing change from baseline to the specified timepoint between conditions. Positive values of $d_{between}$ favour the imagery condition, negative values of $d_{between}$ favour the control condition.

Supplementary Table 2. Results from the mixed model analyses including estimated marginal means and effect sizes for a) Prospective Imagery Test – Positive Vividness b) Scrambled Sentences Test – Negativity.

		a) Prospective Imagery Test – Positive Vividness				
		Baseline	Post-treatment	1-month	3-month	6-month
Intention to treat						
Imagery	*M*	2.81	3.20	3.07	3.05	3.07
(*n* =76)	*SD*	0.88	0.87	0.91	0.87	0.86
	d_{within} [95% CIs]		0.45 [0.23, 0.66]	0.30 [0.07, 0.53]	0.27 [0.06, 0.49]	0.30 [0.06, 0.54]
Control	*M*	2.86	3.03	2.88	2.87	2.82
(*n* =74)	*SD*	0.81	0.86	0.90	0.89	0.87
	d_{within} [95% CIs]		0.21 [0.03, 0.40]	0.03 [-0.18, 0.23]	0.01 [-0.20, 0.23]	0.05 [-0.18, 0.28]
Comparison	*t*(df)		1.86 (142.57)	1.81 (141.72)	1.80 (142.92)	2.17 (142.52)
	p		.07	.07	.07	.03
	$d_{between}$ [95% CIs]		0.31 [-0.02, 0.64]	0.30 [-0.02, 0.63]	0.30 [-0.03, 0.63]	0.36 [0.03, 0.69]
Per protocol						
Imagery	*M*	2.79	3.16	3.06	3.03	3.01
(*n* =59)	*SD*	0.90	0.87	0.91	0.86	0.88
	d_{within} [95% CIs]		0.41 [0.17, 0.64]	0.29 [0.04, 0.55]	0.26 [0.04, 0.49]	0.24 [-0.03, 0.50]
Control	*M*	2.81	2.98	2.84	2.81	2.79
(*n* =56)	*SD*	0.79	0.86	0.90	0.86	0.87
	d_{within} [95% CIs]		0.22 [-0.01, 0.44]	0.04 [-0.21, 0.28]	0.00 [-0.25, 0.25]	0.02 [-0.24, 0.28]
Comparison	*t*(df)		1.49 (113)	1.58 (113)	1.72 (113)	1.48 (113)
	p		.14	.12	.09	.14
	$d_{between}$ [95% CIs]		0.28 [-0.09, 0.65]	0.30 [-0.07, 0.67]	0.32 [-0.04, 0.69]	0.28 [-0.09, 0.65]

		b) Scrambled Sentences – Negativity score	
		Baseline	Post-treatment
Intention to treat			
Imagery	*M*	0.57	0.46
(*n* =76)	*SD*	0.23	0.25
	d_{within} [95% CIs]		0.45 [0.21, 0.69]
Control	*M*	0.60	0.48
(*n* =74)	*SD*	0.24	0.25
	d_{within} [95% CIs]		0.48 [0.24, 0.72]
Comparison	*t*(df)		0.29 (133.49)
	p		.77
	$d_{between}$ [95% CIs]		-0.05 [-0.39, 0.29]
Per protocol			
Imagery	*M*	0.58	0.47
(*n* =63)	*SD*	0.23	0.25
	d_{within} [95% CIs]		0.45 [0.21, 0.70]
Control	*M*	0.58	0.47
(*n* =62)	*SD*	0.25	0.24
	d_{within} [95% CIs]		0.44 [0.20, 0.69]
Comparison	*t*(df)		0.14 (123.00)
	p		.89
	$d_{between}$ [95% CIs]		-0.02 [-0.38, 0.33]

Note. Means, standard deviations, effect sizes and confidence intervals are all derived from the mixed model analysis, with the exception of pre-treatment standard deviation, which is presented as the raw score. d_{within} = within-group effect size for change from baseline to the specified time-point. The *t* and *p* values are for the comparison between conditions of the change from baseline to the specified timepoints (estimates of fixed effects from the mixed model). $d_{between}$ = between-group effect size, comparing change from baseline to the specified timepoint between conditions. Positive values of $d_{between}$ favour the imagery condition, negative values of $d_{between}$ favour the control condition.
